# FAM83D is associated with gender, AJCC stage, overall survival and disease-free survival in hepatocellular carcinoma

**DOI:** 10.1042/BSR20181640

**Published:** 2019-05-07

**Authors:** Xuling Liu, Hong Gao, Jie Zhang, Dongying Xue

**Affiliations:** 1 Department of Infectious Diseases, Putuo Hospital, Shanghai University of Traditional Chinese Medicine, Shanghai 200062, China; 2 Cancer Institute, Putuo Hospital, Shanghai University of Traditional Chinese Medicine, Shanghai 200062, China

**Keywords:** AJCC stage, FAM83D, gender, hepatocellular carcinoma, survival

## Abstract

Prognostic significance of family with sequence similarity 83, member D (FAM83D) in hepatocellular carcinoma (HCC) patients has not been well-investigated using Gene Expression Omnibus (GEO) series and TCGA database, we compared FAM83D expression levels between tumor and adjacent tissues, and correlated FAM83D in tumors with outcomes and clinico-pathological features in HCC patients. Validated in GSE33006, GSE45436, GSE84402 and TCGA, FAM83D was significantly overexpressed in tumor tissues than that in adjacent tissues (all *P*<0.01). FAM83D up-regulation was significantly associated with worse overall survival (OS) and disease-free survival (DFS) in HCC patients (Log rank *P*=0.00583 and *P*=4.178E-04, respectively). Cox analysis revealed that FAM83D high expression was significantly associated with OS in HCC patients [hazard ratio (HR) = 1.44, 95% confidence interval (CI) = 1.005–2.063, *P*=0.047]. Additionally, patients deceased or recurred/progressed had significantly higher FAM83D mRNA levels than those living or disease-free (*P*=0.0011 and *P*=0.0238, respectively). FAM83D high expression group had significantly more male patients and advanced American Joint Committee on Cancer (AJCC) stage cases (*P*=0.048 and *P*=0.047, respectively). FAM83D mRNA were significantly overexpressed in male (*P*=0.0193). Compared with patients with AJCC stage I, those with AJCC stage II and stage III–IV had significantly higher FAM83D mRNA levels (*P* = 0.0346 and *P*=0.0045, respectively). In conclusion, overexpressed in tumors, FAM83D is associated with gender, AJCC stage, tumor recurrence and survival in HCC.

## Introduction

Primary liver cancer, comprising 75–85% cases of hepatocellular carcinoma (HCC), is predicted to be the sixth most commonly diagnosed cancer and the fourth leading cause of cancer death worldwide in 2018 worldwide [1–3]. Although advanced in surgical and nonsurgical therapeutics have been improved over the past decades for the disease, the clinical outcome of HCC remains poor [4] and more than 70% cases developed tumor recurrence at 5 years [5,6]. Hence, the development of novel targeted therapies for HCC treatment requires identification of reliable targets [7,8].

Recently, the family with sequence similarity 83 (FAM83) was shown to have oncogenic potential [9]. A higher expression level of a signature of FAM83 family members was associated with poor prognosis in a number of human cancers [10,11]. In breast cancer, alterations in FAM83 family genes correlated significantly with TP53 mutation and inversely associated with PIK3CA and E-cadherin mutations [9].As a member of FAM83 family, FAM83D is involved in mitotic processes to regulate cell division [12]. Emerging evidence indicated that FAM83D expression is elevated in a wide variety of tumor types including ovarian cancer [13], metastatic lung adenocarcinomas [14] and HCC [15,16], suggesting the possibility that FAM83D is an oncogene for many human malignancies. However, data concerning the expression profiles and clinical impact of FAM83D in HCC patients has not been elucidated.

Our study investigated FAM83D expression levels between tumor and adjacent tissues, and consequently correlated FAM83D in tumors with outcomes and clinico-pathological characteristics in HCC patients, hoping that the data may provide potential biomarker candidates and useful insights into the pathogenesis and progression of HCC.

## Materials and methods

### Source of data

The gene expression data were processed using the RMA algorithm. Gene expression profiles for HCC including GSE33006, GSE45436 and GSE84402 were obtained from Gene Expression Omnibus (GEO) database (https://www.ncbi.nlm.nih.gov/geo/). Tumor and adjacent samples in GSE33006 [17], GSE45436 and GSE84402 [18] were processed on Affymetrix Human Genome U133 Plus 2.0 Array. Affy, AffyPLM and Limma packages in R program were used for quality assessment and identifying FAM83D mRNA expression levels of tumor and adjacent normal samples in each GEO profile. edgeR package was used for identifying FAM83D expression levels in tumor and adjacent tissues in HCC patients.

### Survival analysis

To investigate prognostic significance of FAM83D for predicting the overall survival (OS) and disease-free survival (DFS) of HCC patients, Liver Hepatocellular Carcinoma (TCGA, Provisional) database in cBioPortal for cancer genomics online service was used [19,20]. A z-score threshold ± 2.0 of mRNA expression was selected in genomic profiles and 373 cases with sequenced tumors were conducted for survival analysis.

Additionally, gene data with z scores and clinical data of HCC patients in Liver Hepatocellular Carcinoma (TCGA, Provisional) database were downloaded from cBioPortal and matched with VLOOKUP index in EXCEL, seven hepatocholangiocarcinoma and three fibrolamellar carcinoma cases were excluded, 367 HCC patients were included for further analysis investigating associations between FAM83D and survivals and clinico-pathological features in HCC with FAM83D median cutoff.

### Statistical analysis

The data are presented as mean ± standard deviation (S.D.) or constituent ratio. Differences between the individual groups were analyzed using Student’s *t*-test, χ^2^ test or Ridit analysis. The Kaplan–Meier method was used to compare OS and RFS between different groups, and the log-rank test was used to estimate the difference in survivals. Factors associated with the OS in HCC patients were assessed both by Cox univariate and multivariate analysis. Only covariates significantly associated with outcomes at univariate analysis (two-sided *P*<0.10) included in the multivariate model. Results were reported as hazard ratios (HR) or odd ratios (OR) with 95% confidence intervals (CI). PASW Statistics software version 23.0 from SPSS Inc. (Chicago, IL, U.S.A.) was used. A two-tailed *P*<0.05 were considered significant for all tests.

## Results

### FAM83D expression in HCC patients

**Figure 1:**
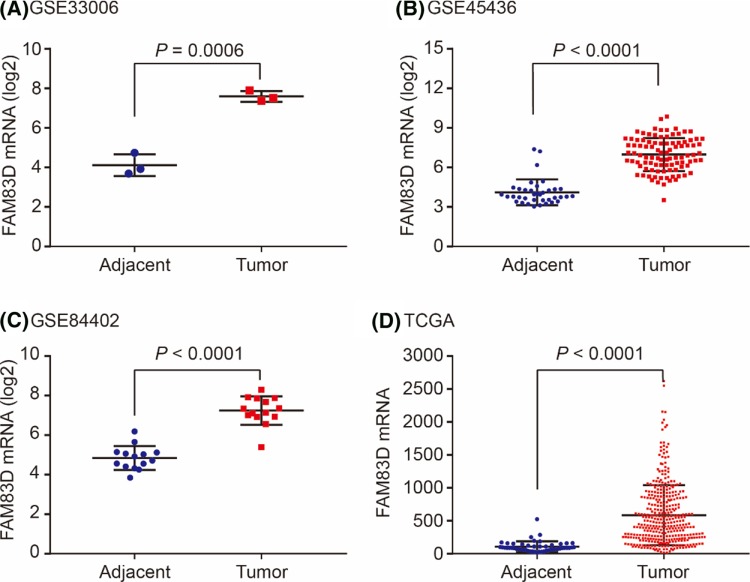
FAM83D expression levels in GEO and TCGA datasets FAM83D expression between tumor and adjacent tissues in GSE33006 (**A**), GSE45436 (**B**), GSE84402 (**C**) and TCGA (**D**).

**Figure 2:**
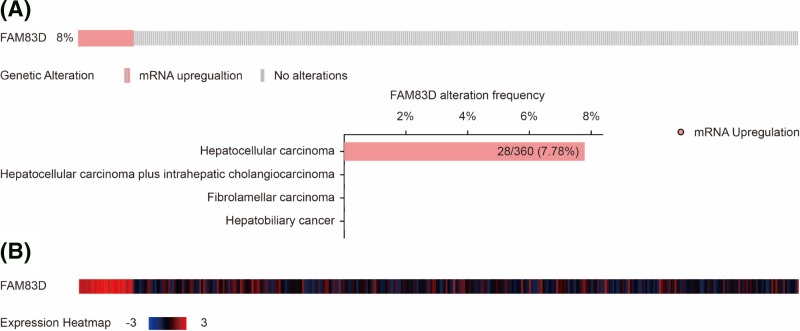
FAM83D alteration and gene expression heatmap in liver cancer FAM83D alteration (**A**) and expression heatmap (**B**) in liver cancer.

The FAM83D mRNA expression levels were calculated in GSE33006, GSE45436 and GSE84402. As shown in Figure 1, FAM83D mRNA were significantly overexpressed in tumor tissues than those in adjacent tissues in the three GEO series (All *P*<0.001, Figure 1A–C). For validation, FAM83D mRNA was also significantly up-regulated in tumors than that in nontumors in HCC patients in TCGA profile (*P*<0.0001, Figure 1D). In addition, we investigate FAM83D alteration distribution in liver cancer. As shown in Figure 2, FAM83D mRNA was up-regulated in approximately 8% HCC patients, no up-regulation of FAM83D mRNA was found in other histological malignancies including HCC plus intrahepatic cholangiocarcinoma, fibrolamellar carcinoma and hepatobiliary cancer (Figure 2).

### Associations between FAM83D and outcomes in HCC patients

**Figure 3:**
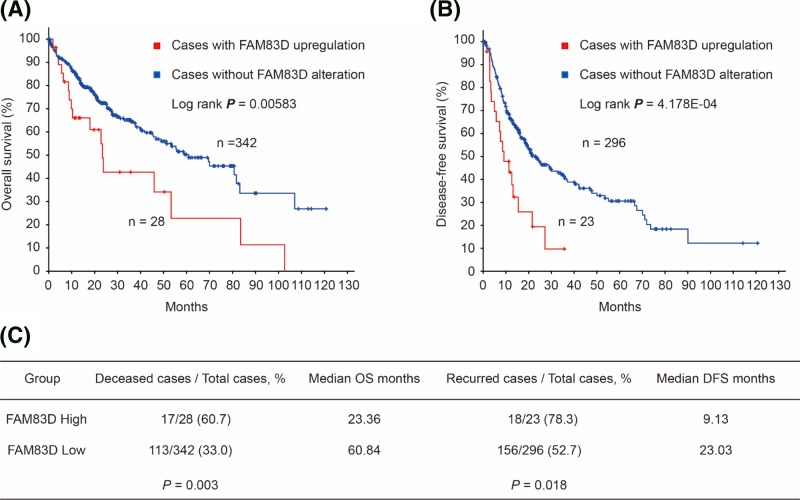
Survival analysis in HCC patients with/without FAM83D alteration OS (**A**) and DFS (**B**) in HCC patients with/without FAM83D alteration.

Using Liver Hepatocellular Carcinoma (TCGA, Provisional) database in cBioPortal for cancer genomics online service, we conducted associations between FAM83D and HCC survival. As shown in Figure 3, FAM83D up-regulation was significantly associated with worse OS (Log rank *P*=0.00583, Figure 3A) and DFS (Log rank *P*=4.178E-04, Figure 3B) in HCC patients. Similarly, the mortality was significantly higher in HCC patients with FAM83D up-regulation than that in cases without alteration (60.7 vs 33.0%, *P*=0.003, Figure 3C). And, HCC patients with FAM83D up-regulation had significantly higher recurrence rate than those without FAM83D alteration (78.3 vs 52.7%, *P*=0.018, Figure 3C).

**Figure 4:**
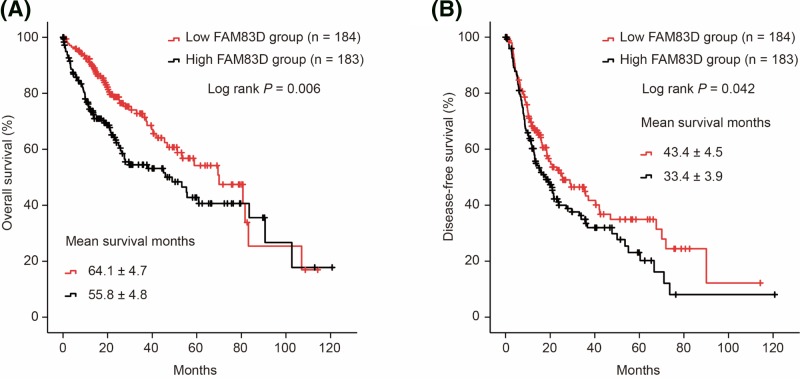
Survival analysis in HCC patients with FAM83D median cutoff OS (**A**) and DFS (**B**) in HCC patients with FAM83D median cutoff.

Moreover, we matched gene data with z scores and clinical data of HCC patients in Liver Hepatocellular Carcinoma (TCGA, Provisional) database with VLOOKUP index. We grouped HCC patients with FAM83D median cutoff. As shown in Figure 4, HCC patients in FAM83D high expression group suffered from significantly poor OS (Log rank *P*=0.006, Figure 4A) and DFS (Log rank *P*=0.042, Figure 4B).

In addition, we performed Cox-regression analysis to investigate the associations between clinico-pathological factors and OS in HCC patients. As shown in Table 2, Univariate-Cox analysis revealed that FAM83D high expression, advanced American Joint Committee on Cancer (AJCC) stage and vascular invasion should be potential risk factors for OS and DFS in HCC patients (all *P*<0.10, Tables 2 and 3). When these factors included in multivariate-Cox regression, FAM83D overexpression and advanced AJCC stage were identified as risk factors for OS in HCC patients (both HR > 1.0 and *P*<0.05, Table 2). And, advanced AJCC stage and macrovascular invasion significantly associated with DFS in HCC patients (both HR > 1.0 and *P*<0.05, Table 3).

### Associations between FAM83D and clinico-pathological features in HCC patients

**Figure 5:**
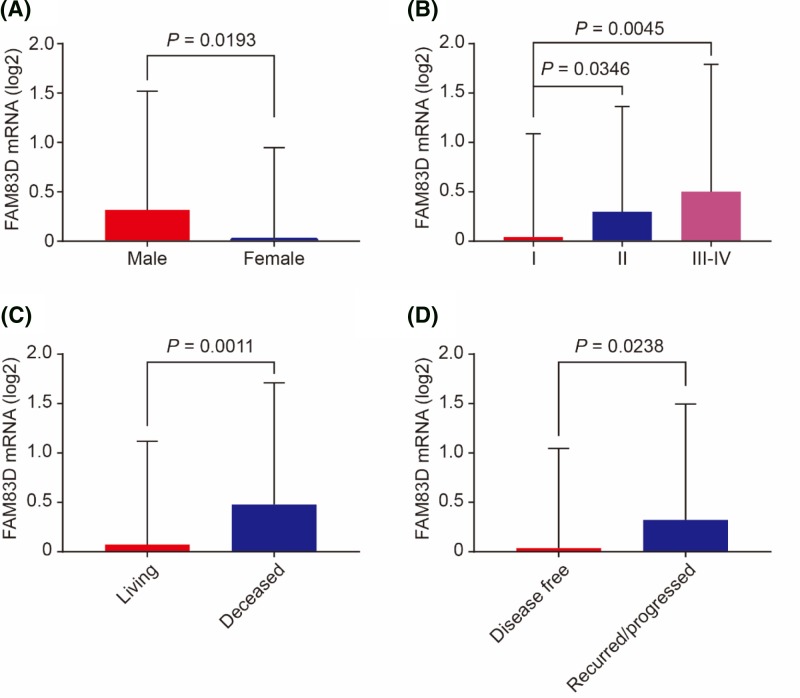
FAM83D expression by gender, AJCC stage and survival status FAM83D expression grouped by gender (**A**), AJCC stage (**B**), overall survival status (**C**) and DFS status (**D**).

Table 1 summarized clinico-pathological features in FAM83D high and low expression groups in HCC patients. FAM83D high expression group had significantly more male cases (*P*=0.048, Table 1). And, HCC patients in FAM83D high expression group suffered from significantly advanced AJCC stage (*P*=0.047, Table 1). Additionally, we compared FAM83D mRNA expression levels grouped by gender, AJCC stage and survival status. We found that FAM83D mRNA were significantly overexpressed in male (*P*=0.0193, Figure 5A). Compared with patients with AJCC stage I, those with AJCC stage II and stage III–IV had significantly higher FAM83D mRNA levels (*P*=0.0346 and *P*=0.0045, respectively, Figure 5B). Consistent with above, patients deceased or recurred/progressed had significantly higher FAM83D mRNA levels than those living or disease-free (*P*=0.0011 and *P*=0.0238, respectively, Figure 5C,D).

**Table 1:** Characteristics of HCC patients between FAM83D high and FAM83D low groups

Variables	FAM83D expression level		*P-*value
	Low (*n*=184)	High (*n*=183)	
Gender, male (%)	116 (63.0)	133 (72.7)	**0.048**
Age, median (IQR), years	61 (17)	61 (19)	0.257
BMI, median (IQR), kg/m^2^	23.9 (7.31)	23.8 (7.31)	0.372
Race, n (%)			0.116
Asian	75 (40.8)	84 (45.9)	
White	96 (52.2)	83 (45.4)	
Black or African American	5 (2.7)	12 (6.6)	
Family history of cancer, n (%)	61 (33.2)	50 (27.3)	0.224
Neoplasm histologic grade, n (%)			
G1–2	122 (66.3)	105 (57.4)	0.065
G3–4	59 (32.1)	76 (41.5)	
AJCC stage, n (%)			**0.047**
I	96 (52.2)	75 (41.0)	
II	37 (20.1)	46 (25.1)	
III	34 (18.5)	51 (27.9)	
IV	3 (1.6)	1 (0.5)	
Vascular invasion, n (%)			0.958
Macro	9 (4.9)	8 (4.4)	
Micro	46 (25.0)	44 (24.0)	
None	108 (58.7)	96 (52.5)	
Child-pugh classification, n (%)			0.574
A	115 (62.5)	103 (56.3)	
B	10 (5.4)	11 (6.0)	
C	1 (0.5)	0 (0)	
AFP > 400 ng/ml, n (%)	28 (15.2)	36 (19.7)	0.261
Platelet, median (IQR), 10^3^/mm^3^	191.5 (144.25)	179 (170)	0.142
New tumor event after initial treatment, n (%)	48 (26.1)	48 (26.2)	0.975
Follow up, median (IQR), days	180 (654)	62 (476)	0.088

IQR, interquartile range; BMI, body mass index; AFP, alpha-fetoprotein.

**Table 2:** Cox regression analysis of risk factors associated with OS in HCC patients

Variables	Univariate		Multivariate	
	HR (95% CI)	*P*-value	HR (95% CI)	*P*-value
FAM83D, log2				
Low	1.0	Reference	1.0	Reference
High	1.624 (1.145–2.304)	0.007	1.44 (1.005–2.063)	**0.047**
AJCC stage				
I	1.0	Reference	1.0	Reference
II	2.727 (1.803–4.124)	<0.001	2.166 (1.369–3.427)	**0.001**
III–IV	2.615 (1.373–4.98)	0.003	2.4 (1.238–4.655)	**0.01**
Vascular invasion				
None	1.0	Reference		
Micro	2.031 (0.967–4.263)	0.061		
Macro	2.702 (1.772–4.121)	<0.001		

All baseline covariates were included in univariable analysis. Only covariates significantly associated with OS in HCC patients at univariable analysis (two-sided *P-*value < 0.10) are shown and included in the multivariable model.

**Table 3:** Cox regression analysis of risk factors associated with DFS in HCC patients

Variables	Univariate		Multivariate	
	HR (95% CI)	*P-*value	HR (95% CI)	*P-*value
FAM83D, log2				
Low	1.0	Reference		
High	1.362 (1.01–1.835)	0.043		
AJCC stage				
I	1.0	Reference	1.0	Reference
II	2.819 (1.961–4.052)	<0.001	2.293 (1.527–3.445)	**<0.001**
III–IV	1.982 (1.069–3.678)	0.03		
Vascular invasion				
None	1.0	Reference	1.0	Reference
Micro	2.042 (1.022–4.079)	0.043		
Macro	2.2 (1.502–3.221)	<0.001	1.555 (1.022–2.367)	0.039

All baseline covariates were included in univariable analysis. Only covariates significantly associated with DFS in HCC patients at univariable analysis (two-sided *P*-value < 0.10) are shown and included in the multivariable model.

## Discussion

As key intermediates in oncogenic EGFR, MAPK, RAS/RAF/MEK/ERK and PI3K/AKT/mTOR signaling, FAM83 involved in a variety of important cancer cell signaling functions and overexpressed in many human cancers [9,10,21–24]. In 17 distinct tumor types, FAM83A, FAM83B and FAM83D most frequently overexpressed in several diverse tissue types [10]. Evidence suggested that elevated expression of FAM83 members is associated with elevated tumor grade and decreased OS [10,21]. Therefore, the FAM83 members are emerging as intriguing oncogenes worthy of additional study.

FAM83D, also known as CHICA, binds to the chromokines in KID and localizes to the spindle during mitosis to regulate spindle maintenance, mitotic-progression and cytokinesis [12,25–27]. Forced expression of FAM83D in nonmalignant cells in culture promoted proliferation and invasion of breast cancer cells and down-regulated the expression of F-box and WD repeat domain-containing 7 (FBXW7), a suppressor of c-Myc, mTOR and C-Jun expression [28]. In colorectal cancer, FAM83D knockdown up-regulated the protein expression level of FBXW7, but diminished the Notch1 protein expression level [29]. As FAM83D regulates tumorigenesis by hyperactivating mTOR, the levels of FAM83D may also predict patient response to rapamycin [28]. The gene amplification and elevated protein expression of FAM83D increased the migration and invasion of breast epithelial cells and was associated with poor prognosis [28,30]. In addition, FAM83D expression was elevated in gastric tumors, and its expression strongly correlated with lymph node metastasis and TNM stage [31]. Exerted its oncogenic activity by regulating cell cycle, FAM83D overexpression is associated with tumor size, lymph node metastases and advanced TNM stage and worse OS in lung adenocarcinoma [32].

In our study, we found that FAM83D was overexpressed in HCC tumors. Patients with advanced AJCC stage had significantly higher FAM83D levels. Interestingly, male patients might be apt to FAM83D elevation compared with female cases. Furthermore, FAM83D elevation in tumors was associated with in worse OS and DFS in HCC patients. The elevation of FAM83D in HCC tumors has been proved previously [15,33]. In our analysis based on TCGA profile, FAM83D mRNA was up-regulated in approximately 8% HCC patients. However, a study by Liao et al. [15] demonstrated that FAM83D was significantly up-regulated in 76.6% of the HCC specimens at the mRNA level and in 69.44% of the HCC specimens at the protein level compared with adjacent noncancerous liver specimens. They also found that FAM83D mRNA expression level was positively correlated with the level of alpha-fetoprotein (AFP), TNM stage, the presence of a portal vein tumor thrombus, OS and DFS time of HCC patients [15,34]. Another report by Lin et al. also indicated that FAM83D overexpression significantly correlated with high HCC recurrence rate after liver transplantation and poor HCC characteristics including high AFP and poor differentiation [33]. In hepatocellular cell lines, FAM83D activates MEK/ERK signaling pathway and promotes the entry into S phase of cell cycle progression [16]. In a xenograft tumorigenesis model, FAM83D knockdown apparently inhibited tumor growth and metastasis [33]. FAM83D promotes HCC recurrence by promoting CD44 expression and CD44^+^ cancer stem cells malignancy via activating the MAPK, TGF-β and Hippo signaling pathways [33]. Consistent with previous publications, we assumed that FAM83D may contribute to hepatocarcinogenesis and constitute a potential therapeutic target in HCC.

In summary, FAM83D may serve as a promising prognostic predictor and therapeutic target for HCC. Up-regulated in tumors, FAM83D is associated with gender, AJCC stage, tumor recurrence and survival in HCC patients. Future research focusing on FAM83D by which FAM83D exerts its oncogenic effects, especially in male population with advanced AJCC stage, requires further clarification.

## Abbreviations

AFP: Alpha-fetoprotein
AJCC: American Joint Committee on Cancer
AKT: Protein kinase B
CI: Confidence interval
DFS: Disease-free survival
EGFR: Epidermal growth factor receptor
ERK: Extracellular regulated MAP kinase
FAM83: Family with sequence similarity 83
GEO: Gene expression omnibus
HCC: Hepatocellular carcinoma
HR: Hazard ratio
IQR: Interquartile range
MAPK: Mitogen activated kinase-like protein
MEK: MAP kinse-ERK kinase
mTOR: Mechanistic target of rapamycin kinase
OS: Overall survival
PI3K: Phosphatidylinositol 3-kinase
RFS: Recurrence free survival
TGF-β: Transforming growth factor beta
TNM: Tumor, Node, Metastases
